# Emotional Regulation, Aesthetic Engagement, and Flow Experience Among Chinese Middle-Aged and Older Choir Members

**DOI:** 10.3390/bs16101842

**Published:** 2026-10-08

**Authors:** Ying Yin, Peiran Xu

**Affiliations:** School of Architecture and Art, Central South University, Changsha 410083, China

**Keywords:** emotional regulation, aesthetic and artistic engagement, intrinsic motivation, flow experience, choral singing, middle-aged and older choir members, structural equation modeling

## Abstract

Choral singing has become a widely practiced collective activity among middle-aged and older adults in China, raising the question of which music-related psychological mechanisms support their deep engagement in this population. Drawing on flow theory and self-determination theory, this study examined how the emotional regulation function and aesthetic and artistic engagement of music were associated with flow experience through intrinsic motivation. A mediation model was tested using structural equation modeling on data from 547 members of Chinese middle-aged and older choirs. Both emotional regulation and aesthetic and artistic engagement were positively associated with intrinsic motivation, which in turn was positively associated with flow experience. Both functions also showed significant direct associations with flow, and showed statistically significant indirect associations with flow via intrinsic motivation. Emotional regulation showed a significantly larger standardized association with intrinsic motivation than aesthetic and artistic engagement (Δβ = 0.306, 95% confidence interval (CI) [0.163, 0.448]); the two functions’ direct associations with flow did not differ significantly (Δβ = 0.057, 95% CI [−0.075, 0.191]). These findings portray music functions and intrinsic motivation as mutually embedded contributors to flow, and contribute to understanding the psychological foundations of musical engagement among middle-aged and older choir members.

## 1. Introduction

Choral singing has become a common collective activity among middle-aged and older adults in China, with members typically attending rehearsals voluntarily and without material reward. While singing, they often become fully absorbed, readily entering a self-forgetting, flow-like state. Music, as an art form that directly engages emotion and attention, has historically been listed among the activities capable of eliciting flow experience (Csikszentmihalyi, 1990). This phenomenon raises a basic question about why music draws this population into such deep engagement.

The specific functions that music serves for these singers may be key to answering this question. Research in this area has developed along several largely separate lines. One line of work has mapped the broad range of purposes that music serves in everyday life, from regulating mood and emotion to supporting social connection and self-reflection (Saarikallio & Erkkilä, 2007; Schäfer et al., 2013). Another line of work has focused on the psychological mechanisms through which music gives rise to emotional responses; mechanisms as different as brain-stem reflexes, evaluative conditioning, emotional contagion, and musical expectancy can each independently produce an emotional reaction (Juslin & Västfjäll, 2008). A further body of work has examined stable individual differences in music preference; preferences for different musical styles reduce to a small number of underlying dimensions that are systematically related to personality traits such as openness to experience (Rentfrow & Gosling, 2003). These three lines of work instead examine, respectively, the functional purposes music serves, the mechanisms of emotional induction, and the structure of individual preference. More recently, it has been proposed that many music-evoked emotions are not well captured by everyday emotion categories such as happiness or sadness (Menninghaus et al., 2019); a class of aesthetic emotions has instead been proposed, including wonder, being moved, and nostalgia, arising from the appreciation of a stimulus’s form and meaning, in contrast to the goal-directed utilitarian emotions studied in most emotion research (Scherer & Zentner, 2001; Timmers et al., 2024; Zentner et al., 2008). Across these functions, this body of work indicates that music can stimulate the intrinsic motivation for deep engagement in musical activity (Engeser & Schiepe-Tiska, 2012; MacIntyre et al., 2018).

Music may draw singers in by offering a channel for regulating and expressing their emotions (Saarikallio & Erkkilä, 2007). Its appeal may also lie in the music itself, in its form and meaning (Menninghaus et al., 2019; Scherer & Zentner, 2001). The present study treats these two routes as two functions of musical activity. Emotional regulation is the use of music to manage present affect. Aesthetic and artistic engagement means attending to the form and meaning of music, and singing that form. Music-emotion research has also distinguished reactions related to personal goals from reactions related to a work’s form (Scherer, 2004). Examined separately, it is not possible to determine how strongly each function is associated with flow, either directly or through intrinsic motivation, or whether those associations differ. Placing both functions within a single model, together with their shared route through intrinsic motivation, addresses this gap.

Choral singing has, at present, become a widespread group musical activity among middle-aged and older adults in China. According to Hunan Provincial People’s Government (2024), in 2023 Hunan Province reported a combined total of 2,595 older-adult choirs and arts groups, with 756,700 older members regularly participating in their activities. The music psychology literature identifies emotional meaning and mood regulation as important reported uses of music among middle-aged and older adults (Hays & Minichiello, 2005; Laukka, 2007). A broader body of work on music and well-being discusses group singing in terms of social connection, emotional support, and meaning-making (Siltainsuu & Peltola, 2024). These reports do not use the same age boundaries. The age range used to define such groups is not consistent in existing writing. Structural equation research on amateur choirs in China has recruited from age 55 and labelled the sample as older adults (Su et al., 2025); local statistics report participation figures under the heading of older-adult choirs (Hunan Provincial People’s Government, 2024). National events named as older-adult choirs also often admit singers from age 50. The Ministry of Culture’s “Forever Glorious” China Older Adults Choir Festival required participating singers to be at least 50 years of age and not older than 75 (Ministry of Culture of the People’s Republic of China, 2017). Middle-aged and older choir activities run by local cultural centres can set an eligibility range from 40 to 65 (Guangming District Cultural Center, 2026). Reviews of adult community choirs often describe members as older, without a unified cutoff (Bloska et al., 2025). Therefore, the specific ages have to be established from the actual social group that was sampled.

To address this question, the present study estimates both functions in one model among Chinese middle-aged and older choir members, with their direct associations with flow and their indirect associations through intrinsic motivation. The comparison tests whether, in a population often described in terms of emotional meaning and mood regulation, aesthetic and artistic engagement remains associated with flow after emotional regulation is taken into account, and which function shows the larger association in this sample.

## 2. Literature Review and Hypotheses

### 2.1. Music Functions

Music function is defined as the psychological effect produced by individuals during musical activities in pursuit of specific goals. The present study draws on the theoretical separation between utilitarian and aesthetic emotions (Scherer, 2004). Utilitarian emotions arise from the appraisal of an event’s relevance to personal goals, needs, or coping potential, and prepare the individual for action (Scherer, 2004; Scherer & Zentner, 2001). Aesthetic emotions, by contrast, arise from the appreciation of the intrinsic qualities of a stimulus, such as its form or structure, and are expressed through diffuse reactions such as shivers or moist eyes (Menninghaus et al., 2019; Scherer & Zentner, 2001).

This study draws on this distinction because it fits well with the specific circumstances of middle-aged and older choral singers. Qualitative and survey research on older adults describes their engagement with music as centering on emotional meaning and mood regulation (Hays & Minichiello, 2005; Laukka, 2007). Within a population whose motivational orientation is often described as dominated by emotional regulation, the utilitarian–aesthetic distinction can be used to judge whether aesthetic and artistic engagement can independently sustain musical engagement.

Emotional regulation function refers to the psychological effect of using musical activity to manage and adjust one’s current emotional state, manifested in the alleviation of tension and anxiety, or in the maintenance of positive emotional experience (Saarikallio & Erkkilä, 2007; Schäfer et al., 2013). This function has long been a central finding in research on the everyday uses of music. Early surveys of listeners’ motivations for musical engagement found that mood management was a commonly reported reason for musical participation (Lull, 1987), a pattern also observed among adolescent listeners (Wells & Hakanen, 1991), and music-related emotional adjustment may also contribute to subjective well-being through the sense of identity, meaning, and belonging that musical participation provides (Ruud, 1997). In the choral context, this function is typically reflected in rehearsal becoming an emotional outlet apart from work or family stress, with members often reporting reduced tension after singing together. The emotional content carried by lyrics and melody is also commonly used as a medium through which individuals process their own emotional experiences (Hays & Minichiello, 2005; Laukka, 2007).

Aesthetic and artistic engagement refers to individuals’ appreciation of and engagement with the form and meaning of music itself, an appreciation that is an end in itself, rooted in attention to the structure, style, and interpretation of a musical work (Menninghaus et al., 2019; Scherer & Zentner, 2001). In the choral context, this function is reflected in sustained attention to harmonic progression and vocal blend, with members noticing the auditory effect of a chord resolving or taking interest in different interpretations of the same passage. In musical activity, technique is often treated as a medium through which artistic expression is realized (Doscher, 1994; Witherspoon, 1925). In choir settings, aesthetic and artistic engagement therefore includes both appreciation of the work and a pursuit of artistic refinement, shown as striving to master musical techniques (Hargreaves, 2012).

Emotional regulation function and aesthetic and artistic engagement thus represent two psychologically distinct pathways of musical participation, the former centered on the present emotional state and the latter on the form and meaning of the music itself (Scherer, 2004). For middle-aged and older choir members, we do not know whether their sustained enthusiasm for singing is better explained by the music’s own form and structure, or by the emotional response this form and structure evokes in the listener. This study takes this as its point of departure, examining the relative contribution of these two functions to intrinsic motivation and flow experience.

From the perspective of self-determination theory, intrinsic motivation is fostered when an activity satisfies the basic psychological needs for autonomy, competence, and relatedness (Deci & Ryan, 2000). Using music to regulate one’s own emotional state is itself a self-directed process (Deci & Ryan, 2000). The individual autonomously turns to music in response to a felt affective need, and doing so successfully can foster a sense of competence in managing one’s own emotional life. Aesthetic and artistic engagement is directed toward appreciation of a work’s form and interpretation, and toward deliberate refinement of technique; motives for refining technique may be intrinsic, extrinsic, or amotivated (Deci & Ryan, 2000). Because each function engages these needs through a different route, both may independently strengthen the intrinsic motivation to continue engaging in musical activity.

**H1.**  *The emotional regulation function of music is positively associated with intrinsic motivation.*

**H2.**  *The aesthetic and artistic engagement of music is positively associated with intrinsic motivation.*

### 2.2. Intrinsic Motivation and Flow

According to self-determination theory, intrinsic and extrinsic motivations can be distinguished, and intrinsic motivation is described as self-determined and spontaneous, arising together with pleasant emotion (Deci & Ryan, 2000). The theory also treats intrinsic motivation as a marker of optimal psychological functioning; longitudinal research in academic settings has linked autonomy-supportive conditions and the satisfaction of basic psychological needs to more sustained engagement over time (Jang et al., 2016).

Flow refers to the state of experiencing “optimal experience” during an activity (Csikszentmihalyi, 1990, 2014). In musical activities, it means being solely focused on music while being indifferent to other things, and feeling extremely pleased with playing music. Flow theory originated from an interest in this kind of self-rewarding, intrinsically motivated activity, and the intrinsic reward that an activity provides is itself regarded as one of the defining features of the flow experience. Entering a flow state, however, also depends on conditions such as the dynamic balance of challenge and skill, clear goals, and immediate feedback, which together direct attention toward the activity in the moment; flow experienced during musical activity has itself been shown to correlate with greater subjective well-being (Loepthien & Leipold, 2022).

Achieving a flow state therefore requires certain prerequisites. A performer confronting music that is extremely challenging relative to their skill level is more likely to feel anxious than immersed. Low challenge paired with low skill tends to produce boredom or apathy instead, and interest in the music fails to develop (Csikszentmihalyi, 1990).

This research on flow indicates that, in addition to being appropriately challenging and requiring necessary skills, musical activities must also sustain performers’ interest over time to avoid boredom. This ongoing interest and willingness to engage is conceptually distinct from the attentional absorption that characterizes the flow state itself. Intrinsic motivation and flow are treated as theoretically distinct constructs; their temporal ordering was not established in this study. In flow theory, the intensity of attention within flow is occasioned by the structural conditions of the activity in the moment (Csikszentmihalyi, 1990).

Consistent with this distinction, prior research has linked this motivational orientation with the occurrence of flow experience. Intrinsic motivation has been found to increase with level of musical activity, from non-musicians to amateurs to professional musicians (Appelgren et al., 2019), and autonomous, intrinsically oriented motivation has been shown to positively predict the experience of flow during musical practice (Valenzuela et al., 2018).

**H3.**  *Intrinsic motivation is positively associated with flow experience.*

Music functions may also have direct associations with flow, apart from this motivational route. Affective and aesthetic engagement with music may immediately change the experience of an activity by regulating arousal, directing attention, or deepening absorption (Juslin & Västfjäll, 2008). Based on empirical research, people who engage with music, even through concentrated listening alone, often report entering a flow state directly, at times to an even greater degree than when performing (Loepthien & Leipold, 2022). An emotionally moving familiar work, for instance, can make a singer lose themselves in the performance for a short period, even when they hold no deep long-term interest in it.

**H4.**  *The emotional regulation function of music is positively associated with flow experience.*

**H5.**  *The aesthetic and artistic engagement of music is positively associated with flow experience.*

Taken together, music functions are associated with intrinsic motivation, and intrinsic motivation, being conceptually distinguishable from the flow state itself, is associated with flow in turn. Intrinsic motivation may therefore play a role in a hypothesized indirect association between music functions and flow, alongside the direct route described above.

**H6.**  *Intrinsic motivation shows an indirect association in the relationship between the emotional regulation function and flow experience.*

**H7.**  *Intrinsic motivation shows an indirect association in the relationship between aesthetic and artistic engagement and flow experience.*

Flow theory specifies conditions such as a balance between challenge and skill, clear goals, and immediate feedback (Csikszentmihalyi, 1990, 2014), and the mechanisms that give rise to flow are correspondingly complex. Research on musical ensembles has linked shared goals, coordination, and social presence among members to group flow (Gaggioli et al., 2017).

Choral singing typically involves concentration from members and regular feedback from the conductor during rehearsal. Amateur choirs also tend to share similar skill levels among members and comparatively unified rehearsal goals, though the present study did not measure these conditions directly. This context motivates our focus on amateur choirs, which allows the study to concentrate on how musical functions and intrinsic motivation relate to flow.

A qualitative study of five community choirs in Victoria, Australia reported that members described social connection, a sense of well-being, and valued experiences of musical participation (Joseph & Southcott, 2018). However, the particular psychological reasons for this special effect are not yet clear. Hallam and Creech (2016) pointed out this problem in their Music for Life project by asking what is special about active music-making that makes it seem to offer benefits beyond those provided by other group activities. This study examines the associations among music functions, intrinsic motivation, and flow experience in choral participation.

**Figure 1:**
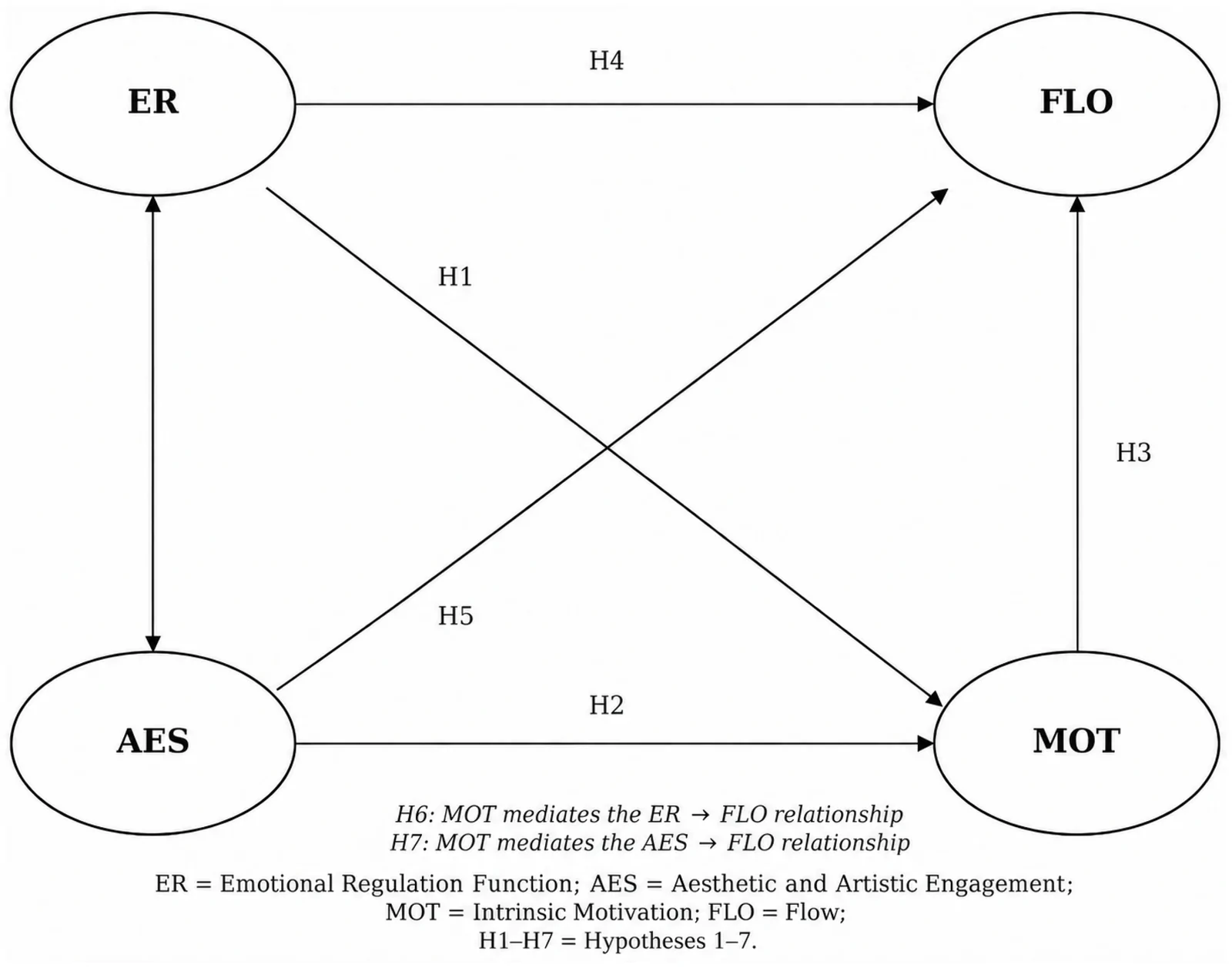
Hypothesis model.

Based on the theoretical synthesis above and the hypotheses developed throughout this section, Figure 1 presents the proposed model linking music functions, intrinsic motivation, and flow experience.

## 3. Method

**Figure 2:**
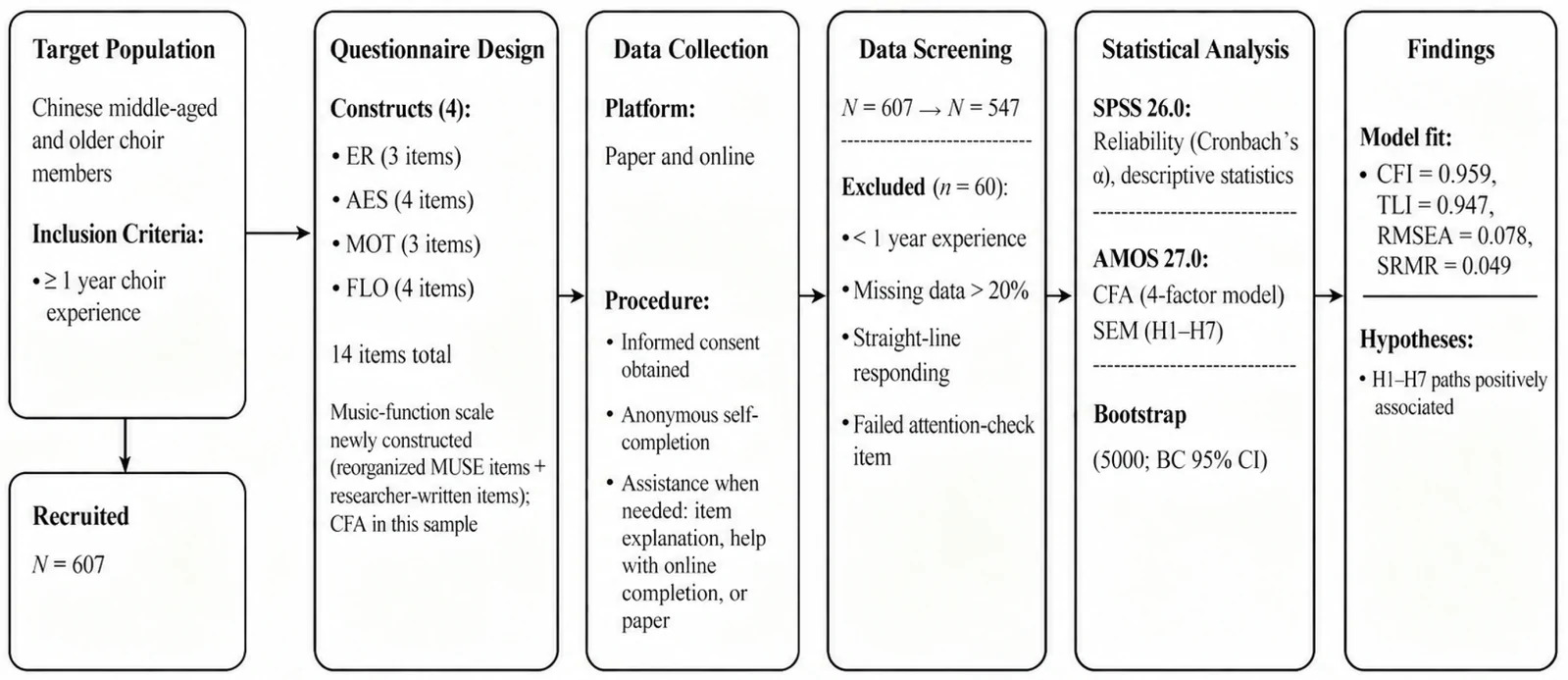
Overview of the research methodology.

Figure 2 provides an overview of the study’s target population, questionnaire design, data collection procedure, data screening steps, statistical analysis, and key findings.

### 3.1. Participants

The sample consisted of amateur middle-aged and older choir members drawn from 10 choir groups across five Chinese cities. The groups were 1 in Shiyan, 2 in Wuhan, and 4 in Yichang, Hubei; 2 in Changsha, Hunan; and 1 in Jining, Shandong. The final analytic sample comprised 547 members. The sampling unit was choirs named as middle-aged and older. Members’ ages are the actual composition of those choirs. The researchers did not set a separate age cutoff. The lowest observed age category was 41–54 years. By questionnaire band, 102 members (18.6%) were aged 41–54 years, 367 (67.1%) were aged 55–64 years, and 78 (14.3%) were aged 65 and older. Members had at least 1 year of choir experience.

### 3.2. Measures

To assess the key constructs, this study adapted instruments commonly used in music and flow research and added researcher-written items.

Music functions were assessed using a custom-developed 7-item scale that combined adapted items from the Music USE (MUSE) questionnaire (Chin & Rickard, 2012) with researcher-developed items. The items were organized according to the two-dimensional structure of music functions adopted in this study, which distinguishes emotional regulation and aesthetic and artistic engagement. The MUSE was selected as the primary source instrument because its empirically validated items have been widely used to assess music engagement in diverse populations. Respondents were instructed to answer all music-function items with reference to their own feelings during choir rehearsals or performances, so that although individual items retained generic wording (e.g., “Music helps me release tension”), responses were anchored to the choral context. However, the original MUSE integrates emotional and cognitive engagement within a single composite style (Cognitive and Emotional Regulation) and does not include items capturing aesthetic dimensions theoretically central to active choral participation, such as stylistic preference and technical-mastery striving. Accordingly, items from the original MUSE were re-organized into two conceptually distinct constructs, and additional items were developed by the research team to capture aesthetic dimensions not represented in the original MUSE item pool. Emotional regulation function (3 items) was measured using items adapted from the MUSE, reflecting the use of music to manage and gain reward from affective experience. Two items were drawn from the Cognitive and Emotional Regulation style (e.g., “Music helps me release tension”; “Music provides an outlet for emotions”). One item was drawn from the Engaged Production style, on the grounds that its affective-reward content (“Performing music is emotionally rewarding for me”) conceptually aligns with the emotion-regulation construct in the context of active musical performance. Aesthetic and artistic engagement (4 items) was measured using two items adapted from the MUSE, capturing inspiration (“Music inspires me”) and reflective engagement (“Music helps me think”). Two additional researcher-developed items captured aesthetic dimensions particularly salient to active choral participation but not represented in the original MUSE item pool, namely stylistic preference (“I like specific styles of music”) and technical-mastery striving (“I strive to master musical techniques”). This operationalization corresponds to the technical-refinement dimension of aesthetic and artistic engagement in Section 2.1. Because the measure combined adapted and newly developed items, its construct coverage should be interpreted cautiously. The hypothesized two-factor structure was supported by confirmatory factor analysis in the main study, and all items loaded on their hypothesized factors above 0.65 (see Section 4.2), with internal consistency (Table 1) and discriminant validity between the two constructs also supported (see Section 3.4).

Intrinsic motivation was assessed using items adapted from the Academic Motivation Scale (Vallerand et al., 1992). Grounded in self-determination theory, the AMS distinguishes three subtypes of intrinsic motivation, namely intrinsic motivation to know (curiosity-driven learning), intrinsic motivation toward accomplishment (mastery and achievement), and intrinsic motivation to experience stimulation (enjoyment of activity participation). The AMS was selected over more general intrinsic-motivation measures (e.g., the Intrinsic Motivation Inventory) because its three-subtype structure aligns particularly well with the experiential nature of musical participation. Choral participation is not a single type of motivation; it includes the wish to learn about music and vocal techniques, a sense of achievement in working with others, and the joy of singing at that moment. These three aspects correspond to the three AMS subtypes, so intrinsic motivation in a musical context maps onto the same three categories. One item was therefore modified for each type. “I seek knowledge through musical participation” (to know) reflects participants’ continuous exploration of repertoire and vocal techniques during rehearsals. “I feel fulfilled through music” (toward accomplishment) refers to a sense of mastery and achievement that comes from performing well and working together in a group. “Singing gives me great pleasure” (to experience stimulation) indicates the emotional joy one feels in singing. Because a short questionnaire was needed, the three items were modelled as indicators of a single latent construct of intrinsic motivation. This approach captures the overall level of intrinsic motivation while retaining theoretical breadth across its three conceptual facets. In the main study (*N* = 547), within the four-factor measurement model, the three intrinsic-motivation items showed substantial standardized loadings on their designated factor. All standardised factor loadings exceeded 0.70, with two of the three above 0.80 (see Section 4.2), internal consistency was good (Table 1), and discriminant validity between intrinsic motivation and other constructs was supported (see Section 3.4). The three motivation items do not measure temporal stability or intention to continue participating.

Building on Csikszentmihalyi’s (1990) theory of flow, Jackson and Marsh (1996) developed the nine dimensions of the Flow State Scale to operationalize flow; three of these dimensions, challenge–skill balance, clear goals, and immediate feedback, are regarded as the necessary preconditions for entering a state of flow, and the other six dimensions describe the experience of being in a state of flow. Following Su et al. (2025), this study treated the three prerequisite dimensions as antecedents to flow, distinct from flow itself, and consolidated the six experiential dimensions into four core indicators of the flow experience. Flow was therefore assessed using four single-item indicators, each corresponding to one core experiential dimension of flow in choral singing, namely concentration (“completely focused on singing”), absorption (“losing track of time”), sense of control (“being in control of the performance”), and enjoyment (“finding singing enjoyable”). Based on Su et al.’s (2025) findings, the wording of the items has been revised to be more specific and easier to understand. A simplified design was employed to reduce the response burden, while maintaining the theoretical coverage of the main flow experience. Confirmatory factor analysis of the main study (*N* = 547) supported the unidimensional structure of the 4-item flow scale, with all standardized factor loadings exceeding 0.80 (see Section 4.2). Internal consistency was good (Table 1), and discriminant validity was also supported (see Section 3.4). Intrinsic motivation and flow are treated as theoretically distinct constructs; their temporal ordering was not established in this study. Although both constructs may involve positive affect, the items were selected to represent these distinct theoretical levels.

The questionnaire was administered in Chinese. Supplementary Table S1 presents the Chinese items used in the survey alongside the English wording reported in the manuscript, with the source and adaptation status specified for each item.

The Chinese items were translated and adapted jointly by a researcher and a research assistant, both of whom work in music research and read the source instruments in English. The Chinese items are adaptations and are not literal translations of the English source wording.

Before distribution, a retired professor of choral music completed the questionnaire and commented on the wording. Following those comments, wording that referred to musical activity in general was made specific to the choral setting where this aided comprehension. The emotional regulation item drawn from the Engaged Production style names choral performance in the administered Chinese, where the source instrument refers to performing music. The aim was to make the items easier for middle-aged and older choir members to understand. After these revisions, the same two people translated the Chinese items back into English and compared the result with the source wording. No record of this back-translation, or of the wording changes made after the professor’s comments, was retained. Cognitive interviewing with members of the target population was not conducted. The administered wording of every item is given in Supplementary Table S1.

The two researcher-written aesthetic items were developed alongside the adapted items and were included in the same questionnaire pretest. Aes4 addresses striving to master musical technique, whereas Aes3 addresses preferences for particular musical styles.

### 3.3. Procedure

Participant recruitment took place through direct engagement with amateur middle-aged and older choir groups. After the conductor or choir leader agreed, the questionnaire was administered on paper and online after rehearsal, following the tailored design method for anonymous surveys proposed by Dillman (2011). Assistance consisted of explaining items, helping with online completion, or providing a paper version. Assistants did not complete attitude items for respondents. In total, 607 responses were received, of which 547 met the validity criteria described below. To minimize disruption to rehearsals, participants completed the questionnaires after their regular rehearsal sessions. The data screening followed a multi-step process. Of the 607 responses received, 11 were excluded because respondents reported fewer than one year of choral experience, 4 because more than 20% of item responses were missing, 26 because they failed a single attention-check item embedded in the questionnaire, and 19 because of mechanical straight-line responding, defined as identical responses across all items, including the attention-check item. These exclusions yielded a final analytic sample of 547 participants, none of whom had missing data on the 14 items used in the reported analyses. Skewness ranged from −1.60 to −0.08 across items, and kurtosis ranged from −1.33 to 2.65; both fall within the range generally treated as acceptable for maximum-likelihood estimation (Kline, 2023), supporting the suitability of the data for parameter analysis.

The procedures followed the principles of the Declaration of Helsinki regarding informed consent. All participants were informed of the study’s purpose and procedures and of their rights as participants before taking part, and voluntarily provided informed consent. All questionnaires and tests were carried out anonymously, and all data were only used for academic research purposes. The research team stored all materials safely to ensure data security and the protection of participants’ privacy.

### 3.4. Data Analysis

For descriptive statistics and demographic comparisons, construct scores were calculated as the arithmetic mean of their constituent items. Structural equation analyses used the individual items as indicators of latent constructs.

This study used SEM (Structural Equation Modeling) as the main analytical framework to explore the multivariate associations among latent variables. SEM allowed the relationships among emotional regulation function, aesthetic and artistic engagement, intrinsic motivation, and flow to be assessed simultaneously, along with the fit of the overall model.

Through CFA (Confirmatory Factor Analysis) carried out with AMOS 27.0 using maximum-likelihood estimation, the structural validity of the measurement model is further verified. The results show that the chi-square ($\chi^{2}$) value is 305.397, the degrees of freedom (df) are 71, and the ratio of chi-square to degrees of freedom ($\chi^{2}$/df) is 4.301. This ratio meets the acceptable range proposed by Kline (2023). Other fitting indices, such as IFI (Incremental Fit Index = 0.959), TLI (Tucker–Lewis Index = 0.947), and CFI (Comparative Fit Index = 0.959), all exceed conventional thresholds (Kline, 2023), while RMSEA (Root Mean Square Error of Approximation = 0.078) falls in a range more commonly described as acceptable than as close fit (Browne & Cudeck, 1992). Taken together, the indices support a model with adequate, though not unequivocally close, fit (Table 2).

A bias-corrected bootstrap procedure with 5000 resamples and 95% confidence intervals (CIs) was used to examine indirect associations, in order to obtain robust estimates of indirect effects and avoid assuming normality (Preacher & Hayes, 2008). The same bias-corrected bootstrap procedure was used to test pairwise differences in standardized path coefficients. For each of 5000 resamples, the model was refitted, standardized estimates were obtained, and bias-corrected 95% confidence intervals were constructed for each difference (Δβ).

Further examination of discriminant validity showed that the square root of the Average Variance Extracted (AVE) for each construct exceeded its highest correlation with any other construct (Table 3). A heterotrait–monotrait ratio (HTMT) analysis was also conducted for all six construct pairs (ER–AES = 0.223, ER–MOT = 0.641, ER–FLO = 0.454, AES–MOT = 0.387, AES–FLO = 0.354, MOT–FLO = 0.530). All values were below 0.85, supporting construct-level discriminant validity.

After review, the 14-item model was re-estimated after Er3 was dropped, after Aes4 was dropped, and after both items were dropped. These models are reported as post hoc sensitivity analyses. The primary model retains all 14 items. Standardized estimates for all four models are given in Section 4.4 and in Supplementary Table S4.

## 4. Results

### 4.1. Descriptive Statistics

Table 4 presents the demographic characteristics of the 547 valid participants. Independent-samples t tests (pooled variance, df = 545) found no significant gender differences on the four item-mean composites, all |t|(545)≤1.44, p≥0.151, |d|≤0.15. One-way analysis of variance (ANOVA) found no significant age-band differences on the same composites, largest F(2,544) = 1.50, *p* = 0.225, $\eta^{2}$ = 0.005. For education, ER, AES, and MOT did not differ. FLO differed by education, F(6,540) = 2.46, *p* = 0.023, $\eta^{2}$ = 0.027. The full 12-cell statistics are in Supplementary Table S3.

Means and standard deviations (5-point Likert scale) were as follows. Flow had a mean of 3.394 (SD = 1.216), intrinsic motivation 4.201 (SD = 0.827), emotional regulation 4.116 (SD = 0.938), and aesthetic and artistic engagement 3.567 (SD = 0.964).

The sample came from 10 choirs. Members of the same choir share rehearsal conditions, conductors, and repertoire, so responses may cluster. The structural equation model treated the 547 respondents as independent observations and did not adjust standard errors or significance tests for clustering.

### 4.2. Measurement Model Results

Internal consistency for each construct was first assessed using Cronbach’s alpha, which ranged from 0.858 to 0.936 across the four constructs (Table 1). In the evaluation of the measurement model, Composite Reliability (CR) and Average Variance Extracted (AVE) were also computed (Table 5). All the CR values exceeded the Hair et al. (2013) threshold of 0.70, and all the AVE values exceeded the Fornell and Larcker (1981) threshold of 0.50. Both reliability and convergent validity were therefore acceptable.

### 4.3. The Structural Model

The structural component of the model was saturated. The freely estimated covariance between ER and AES, together with the two regression paths to MOT and the three regression paths to FLO, parameterized all six relationships among the four latent constructs. Accordingly, the structural model imposed no additional covariance restrictions beyond the measurement model and reproduced its fit statistics exactly. These fit indices therefore evaluate the measurement model. They do not separately test directional structural relations. Alternative structural orderings that are equally saturated could reproduce the same covariance matrix; the present data therefore do not adjudicate the hypothesized causal direction. The model showed fit in the acceptable range, short of close fit. $\chi^{2}$ = 305.397 (df = 71, $\chi^{2}$/df = 4.301), IFI = 0.959, TLI = 0.947, CFI = 0.959, SRMR = 0.049, and RMSEA = 0.078 (90% CI [0.069, 0.087]). Most indices met conventional guidelines (Kline, 2023), and the RMSEA value falls in a range conventionally described as acceptable, not close fit (Browne & Cudeck, 1992); the RMSEA confidence interval does not support a claim of close fit.

**Figure 3:**
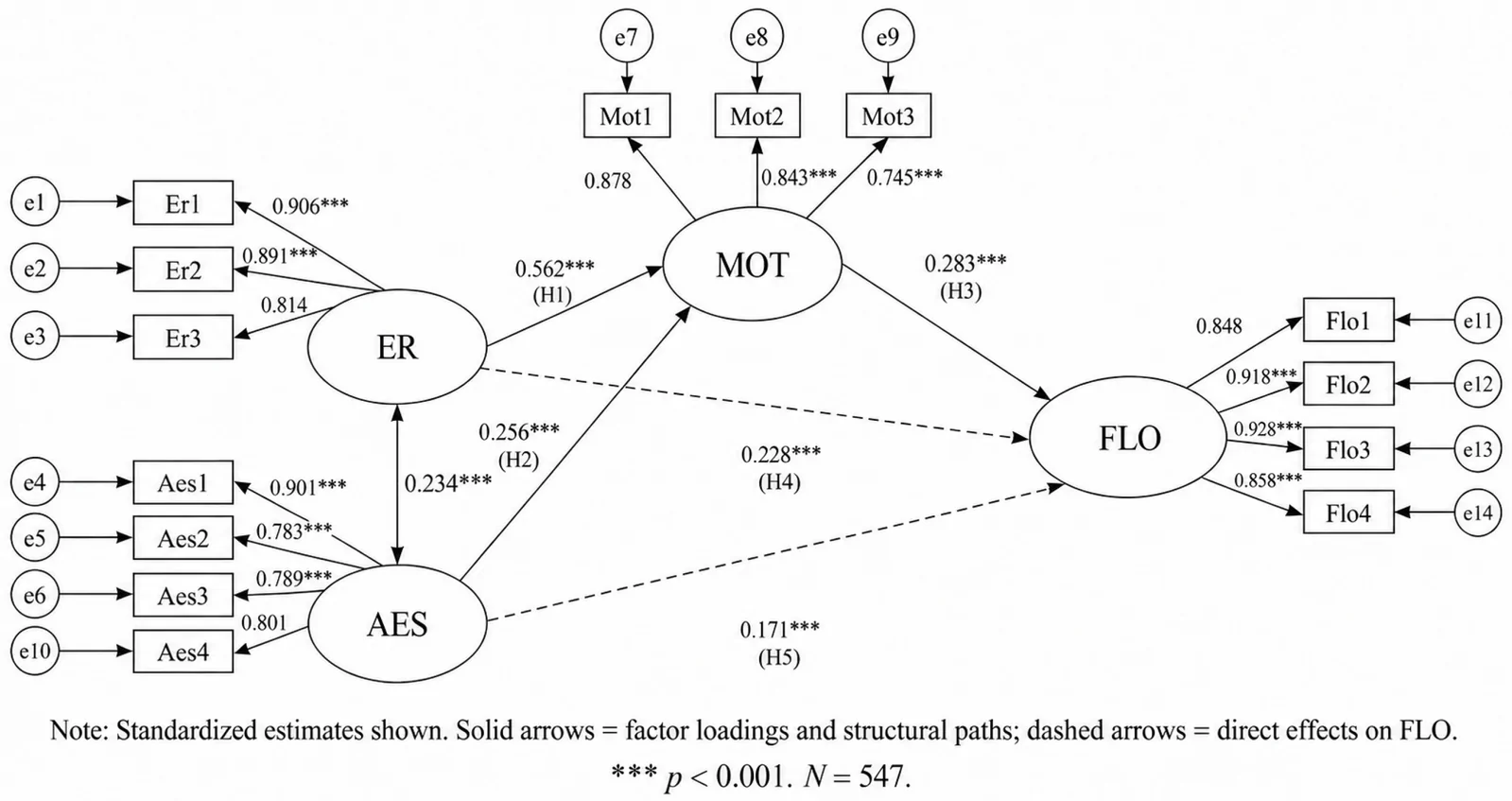
Estimates of the structural equation model.

Structural path coefficients are reported in Table 6, and the two indirect associations in Table 7. All square roots of AVE were higher than the corresponding inter-construct correlations, supporting discriminant validity. Figure 3 presents the full set of standardized parameter estimates for the structural equation model, including factor loadings and structural paths.

The specified model accounted for 44.8% of the variance in intrinsic motivation ($R^{2}$ = 0.448) and 29.7% of the variance in flow experience ($R^{2}$ = 0.297).

The H1–H5 paths were positive and significant (Table 6). Emotional regulation function was positively associated with intrinsic motivation (H1; β = 0.562, C.R. = 12.623, *p* < 0.001), as was aesthetic and artistic engagement (H2; β = 0.256, C.R. = 6.250, *p* < 0.001). Intrinsic motivation was positively associated with flow experience (H3; β = 0.283, C.R. = 4.747, *p* < 0.001). Both music functions also showed significant direct associations with flow after accounting for intrinsic motivation, including emotional regulation (H4; β = 0.228, C.R. = 4.169, *p* < 0.001) and aesthetic and artistic engagement (H5; β = 0.171, C.R. = 3.846, *p* < 0.001). Music functions showed significant direct associations with flow and, as reported below, significant indirect associations with flow via intrinsic motivation.

To test H6–H7, a bias-corrected bootstrap procedure with 5000 resamples and 95% confidence intervals was conducted. Emotional regulation showed a statistically significant indirect association with flow via intrinsic motivation (β = 0.159, 95% CI [0.090, 0.235]), as did aesthetic and artistic engagement (β = 0.072, 95% CI [0.038, 0.114]; see Table 7). Neither confidence interval included zero. The direct associations and the indirect associations via intrinsic motivation were both statistically significant.

### 4.4. Sensitivity Analyses

The primary analysis is the 14-item model. In the primary model, the standardized path difference toward intrinsic motivation was 0.306 (95% CI [0.163, 0.448]), whereas the difference between the direct paths to flow was 0.057 (95% CI [−0.075, 0.191]). After review, three reduced models were estimated (Supplementary Table S4). After Er3 was dropped, ER → MOT changed from 0.562 to 0.543 (bias-corrected 95% CI [0.454, 0.627]), and the path difference toward intrinsic motivation still excluded 0 (Δβ = 0.296, 95% CI [0.145, 0.449]). After Aes4 was dropped, AES → MOT changed from 0.256 to 0.248 and AES → FLO from 0.171 to 0.149; both remained significant. When both items were dropped, H1–H7 remained significant. Across all four models, the path difference toward intrinsic motivation excluded 0. The difference between the two functions’ direct paths to flow included 0.

## 5. Discussion

This study examined how the emotional regulation function of music and aesthetic and artistic engagement were associated with the experience of flow among Chinese middle-aged and older choir members, and whether intrinsic motivation showed a statistically significant indirect association in this relationship. The results indicate that music functions and intrinsic motivation were jointly associated with the flow experience. Music’s affective and aesthetic engagement, intrinsic motivation, and flow experience appear to form an interconnected psychological system.

### 5.1. Music Functions and Intrinsic Motivation as Joint Contributors to Flow

The associations between music functions and flow are not fully explained by intrinsic motivation. Both emotional regulation (β = 0.228, *p* < 0.001) and aesthetic and artistic engagement (β = 0.171, *p* < 0.001) were significantly associated with flow even after accounting for the indirect association via intrinsic motivation. Intrinsic motivation alone does not account for this association, and music functions retain an independent link to flow.

This pattern is consistent with Csikszentmihalyi’s (2014) account of flow, in which the structural conditions of an activity, namely a workable balance between challenge and skill, clear proximal goals, and immediate feedback, direct attention toward the activity from moment to moment, with the intrinsic reward this generates treated as one of flow’s own defining features. Music functions may plausibly supply some of this structural and affective material within choral singing, although the present study did not measure these prerequisite conditions directly. The emotional cues and aesthetic qualities of a piece appear linked to the moment-to-moment attentional engagement associated with flow, and intrinsic motivation appears linked to this same engagement.

Aesthetic and artistic engagement retained a significant, independent association with flow after emotional regulation and intrinsic motivation were entered into the same model. This is consistent with treating aesthetic and artistic engagement, as operationalized in the present study, as an independently associated aspect of musical experience within this choral context.

### 5.2. Path Difference Toward Motivation

Of the two music functions examined in this sample, emotional regulation showed the larger association with intrinsic motivation. The difference between the two functions’ direct paths to flow was not statistically significant (Section 4.4). The interpretations that follow therefore concern a difference specific to the motivational pathway. The stronger association of emotional regulation in this study is in line with previous research; older adults have also turned to music for mood management and life enhancement (Hays & Minichiello, 2005; Laukka, 2007). One account of this pattern draws on socioemotional selectivity theory. This theory holds that when perceived time horizons narrow, whether through aging or other constraints, motivational priorities change (Carstensen, 1992, 2006). Because it concerns motivational priorities, it maps more directly onto the significant path difference toward intrinsic motivation. The present sample did not measure perceived time horizon, retirement, or role transition, and it has no younger comparison group, so the observed pattern cannot be tested as the motivational process proposed by that theory. The theory is offered here only as an interpretation that still needs a direct test.

### 5.3. Aesthetic and Artistic Engagement as an Independently Associated Contributor

The relatively weaker estimated associations of aesthetic and artistic engagement should not be read as showing that it is unimportant in this sample. It was nonetheless significantly associated with both intrinsic motivation and flow. Meaning-oriented appreciation and stylistic engagement therefore remain meaningful psychological contributors to the choral experience. Emotional regulation and aesthetic and artistic engagement are therefore best regarded as related but empirically distinguishable contributors, consistent with how the two constructs were operationalized and modeled in this study. In this sample, the two showed distinct patterns of association with intrinsic motivation and flow.

### 5.4. Theoretical Reflections

Together, these results deepen our understanding of flow in music. Consistent with Csikszentmihalyi’s (2014) distinction between the structural antecedents of flow and the intrinsic reward that flow itself provides, the present findings show that intrinsic motivation, although theoretically relevant, was not the only variable associated with flow in this sample. The findings are consistent with music-related emotional and aesthetic engagement being associated with flow alongside intrinsic motivation.

## 6. Potential Implications and Directions for Intervention Research

The results of this study have some implications for choral practice, music education, and arts-based interventions for middle-aged and older adults. Music functions and intrinsic motivation are linked psychological processes during choral participation, but their relative importance need not stay fixed. It may shift as participation continues and technical ability develops, and it may vary across rehearsal contexts. These associations, taken at one time point, offer tentative hypotheses for choral practice, which require prospective or experimental evaluation before they can inform practice recommendations.

The present data, collected at one time point, cannot show whether the relative weight of emotional and aesthetic engagement shifts as participation continues. Cultivating aesthetic engagement may be valuable, but the present findings offer no basis for treating it as something that displaces affective engagement. Emotional regulation was positively associated with flow experience, so it may remain one way in which singers are psychologically supported during rehearsal. Music can serve an emotion-regulatory function for some older adults (Hays & Minichiello, 2005; Laukka, 2007). A practice-oriented approach may seek to accommodate both emotional and aesthetic engagement. Subsequent work can control emotional and aesthetic conditions separately during rehearsal and compare whether engagement and flow are maintained.

Across the findings of this study, intrinsic motivation was consistently associated with music functions and flow, in line with the specified indirect association reported above. In practice, choral rehearsal may benefit from supporting members’ intrinsic motivation to learn music for its own sake, alongside its usual focus on performance outcomes (Deci & Ryan, 2000; Evans & Liu, 2019). These suggestions are hypotheses for future intervention research. Conductors might give members choices in repertoire, acknowledge their progress in both music and life, and frame feedback around learning and growth instead of grades (Jang et al., 2016). This recommendation is consistent with recent evidence from university music students, where instructors’ autonomy-supportive teaching predicted students’ autonomous motivation, which in turn predicted both the amount and the quality of practice, while controlled motivation showed no such association (Bonneville-Roussy & Evans, 2025). Whether this pathway extends to middle-aged and older community choir conductors and their members remains to be tested. Intrinsic motivation cultivated in this way may allow individuals to integrate feeling and aesthetics into their broader musical participation.

Accessible group singing activities such as community choirs may offer opportunities for emotional regulation, social interaction, and belonging. Whether programmes designed around these processes serve public well-being, or sustain long-term interest in music, was not examined here. Both remain questions for future research.

## 7. Limitations and Future Directions

This study specified and tested a structural model connecting music functions, intrinsic motivation, and flow experience among Chinese middle-aged and older choir members. Although the estimated associations were consistent with the hypothesized pattern, the study does not establish the proposed causal ordering. Several limitations should be acknowledged; at present, these are issues of interpreting the results and future research directions. This study was not preregistered, and no a priori sample-size or power calculation was conducted. The back-translation was carried out by the same two people who prepared the Chinese items, and no record of it was retained. Semantic equivalence between the Chinese items and the English source instruments was therefore not independently verified. Cognitive interviewing with members of the target population was not conducted, which limits the evidence available on how participants understood the adapted items. Health and anxiety were not assessed, and continued participation was not tracked, so the findings do not establish effects on physical or mental health, retention, or dropout.

Most of the choir participants in this study were women (78.1%), and although the sample was drawn from several regions of China, all participants belonged to the same national and cultural context. This gender disparity and the confinement of the sample to China limit the generalisation of the results to male choir members or to choral populations in other cultural settings. This gender imbalance is not unique to the present sample; a recent scoping review notes that prior literature commonly describes adult community choirs as having older and predominantly female memberships (Bloska et al., 2025), suggesting that the pattern observed here is not an isolated feature of this sample. Future research building on this study could collect data from different areas of China and abroad to improve the generalisability of the model. The sample comprised active, continuing volunteers with at least one year of choir experience; the findings may not generalize to individuals who never joined or who discontinued participation, and cannot establish whether music functions are associated with entry into or retention in choir activity.

All variables were measured at one time point, so the present study could not establish the causal ordering of music functions, intrinsic motivation, and flow. Although the directions of the paths tested in this study follow flow theory and self-determination theory (Csikszentmihalyi, 1990; Deci & Ryan, 2000), reciprocal or other causal relationships cannot be ruled out empirically. Follow-up research tracking choir members at different points during rehearsal, or experimentally altering other aspects of the music, could offer more direct evidence for the direction of these relationships.

The sample was drawn from 10 choir groups, and members within the same choir likely share rehearsal conditions, conductors, and repertoire that could induce within-group correlation among responses. The structural equation model reported here treated all 547 respondents as independent observations and did not adjust the standard errors or significance tests for this clustering. If members of the same choir resemble one another more than members of different choirs do, the reported standard errors may be too small and the associated significance levels too optimistic. Future work should record choir membership and report the intraclass correlation of key variables, using cluster-robust standard errors or multilevel models where indicated.

Several measurement-related considerations should also be noted. The music-function scale in this study combined adapted items from the Music USE questionnaire (Chin & Rickard, 2012) with researcher-developed items designed to cover aesthetic attributes of choral participation. The empirical results show a two-factor structure with good reliability and validity, but further research is needed to develop fully validated music-function measures and confirm these findings. A related concern applies specifically to the aesthetic and artistic engagement items, which combined stylistic engagement (“Music inspires me,” “Music helps me think,” “I like specific styles of music”) with a technical-mastery item (“I strive to master musical techniques”). This last item may reflect competence striving or general participatory investment, and it may be conceptually adjacent to the accomplishment-oriented items used to measure intrinsic motivation. The single-factor confirmatory model supports covariation among these items, and the Fornell–Larcker comparisons reported above support discriminant validity between this construct and the other constructs. Neither check can establish that these items jointly measure a unified aesthetic construct. This possibility should be considered when interpreting the aesthetic and artistic engagement path estimates. A related limitation concerns the flow scale, which was realised as four single-item indicators of the core experiential dimensions, following the simplified method introduced by Su et al. (2025). This design reduced the response burden, but it also lowered the psychometric reliability of assessing individual flow dimensions, so multi-item flow measures could provide more detailed data in future research where cognitive load permits. Because all variables were self-reported, the study also remains subject to common-method bias and social desirability bias (Podsakoff et al., 2003). Emotional regulation, aesthetic and artistic engagement, intrinsic motivation, and flow were all measured within a single session using conceptually related items. This bias may therefore take the specific form of consistency-motivated responding, in which participants who perceive these constructs as related tend to generate compatible answers across them (Podsakoff et al., 2003). A recent review of method-bias sources and remedies discusses this mechanism further (Yao & Xu, 2024). Because questionnaires were completed after regular rehearsals, covariance among the self-reports may also partly reflect transient rehearsal-specific affect or experience. Future work could address this limitation by supplementing self-report with conductor ratings or physiological indicators of flow, or by separating the measurement of these constructs in time. Although we have explained the intended distinctions among the items at the construct-design level, respondents may still have been unable to fully separate what the two items referred to when they answered. The emotional regulation item drawn from the Engaged Production style, “Performing music is emotionally rewarding for me,” shares affective wording with the intrinsic motivation items “I feel fulfilled through music” and “Singing gives me great pleasure.” The confirmatory factor analysis, the Fornell–Larcker comparisons, and the HTMT ratio reported above (0.641 for this pair, the highest among all construct pairs though still below 0.85) all supported discriminant validity between the two constructs at the scale level. None of these checks, however, can rule out that this item-level wording proximity inflated the estimated association between emotional regulation and intrinsic motivation. After Er3 was dropped, ER → MOT changed from 0.562 to 0.543 (bias-corrected 95% CI [0.454, 0.627]); the path difference to MOT still excluded 0. A further item-level overlap concerns intrinsic motivation and flow. Mot3 (“Singing gives me great pleasure”) and Flo4 (“finding singing enjoyable”) both refer to pleasure in singing. The reported sensitivity analyses dropped Er3 and Aes4; they do not test whether this wording proximity contributed to the MOT → FLO association.

Finally, although music functions and intrinsic motivation were examined as correlates of flow in this paper, other factors that can affect flow in choral settings have not been included, such as group cohesion, conductor’s leadership type, selection of music works, individual characteristics, or musical background. Future research will incorporate these contextual and individual-difference variables to explore the phenomenon of flow in group musical activities more broadly.

## 8. Conclusions

This paper proposed and tested a structural model of musical functions, intrinsic motivation and flow experience for middle-aged and older choir members in China. Both emotional regulation and aesthetic and artistic engagement were positively associated with intrinsic motivation, and intrinsic motivation, in turn, was positively associated with flow. Both music functions were associated with the flow experience.

These results suggest that music functions and intrinsic motivation are interwoven in their association with the flow experience for middle-aged and older choir members. The affective and aesthetic engagement that music stimulates was associated with intrinsic motivation, which was in turn associated with flow. Emotional regulation showed a significantly larger association with intrinsic motivation than aesthetic and artistic engagement in this sample, though the two functions’ direct associations with flow did not differ significantly. Aesthetic engagement with the choral repertoire was nonetheless independently associated with flow.

Taken together, musical engagement is associated with the psychological experience of participation in community choirs. The present sample includes only members who were still in a choir and who had sung for at least one year, and the questionnaire did not measure health. Whether supporting intrinsic motivation helps sustain affective and aesthetic involvement, and whether choir participation is related to continued participation or physical or mental health, still needs longitudinal or experimental work. The present results do not extend to those outcomes.

## Figures and Tables

**Table 1:** Reliability analysis.

Construct	Cronbach’s α	Items
ER (Emotional Regulation Function)	0.903	3
AES (Aesthetic and Artistic Engagement)	0.890	4
MOT (Intrinsic Motivation)	0.858	3
FLO (Flow)	0.936	4

Note: ER = Emotional Regulation Function; AES = Aesthetic and Artistic Engagement; MOT = Intrinsic Motivation; FLO = Flow.

**Table 2:** Model fit summary.

$\chi^{2}$	df	$\chi^{2}$/df	GFI	CFI	IFI	NFI	TLI	SRMR	RMSEA [90% CI]
305.397	71	4.301	0.920	0.959	0.959	0.947	0.947	0.049	0.078 [0.069, 0.087]

Notes: $\chi^{2}$ = chi-square; df = degrees of freedom; $\chi^{2}$/df = chi-square divided by degrees of freedom; GFI = Goodness-of-Fit Index; CFI = Comparative Fit Index; IFI = Incremental Fit Index; NFI = Normed Fit Index; TLI = Tucker–Lewis Index; SRMR = Standardized Root Mean Square Residual; RMSEA [90% CI] = Root Mean Square Error of Approximation with 90% confidence interval. As noted in the text, the structural model is saturated relative to the measurement model, so these indices evaluate the measurement model.

**Table 3:** Descriptive statistics and discriminant validity.

Constructs	AES	ER	MOT	FLO	M	SD
AES	0.820				3.567	0.964
ER	0.234	0.871			4.116	0.938
MOT	0.387	0.622	0.824		4.201	0.827
FLO	0.334	0.444	0.491	0.889	3.394	1.216

Note: ER = Emotional Regulation Function; AES = Aesthetic and Artistic Engagement; MOT = Intrinsic Motivation; FLO = Flow. Diagonal values are the square roots of the Average Variance Extracted (AVE). M and SD are the mean and standard deviation of the arithmetic item-mean scores described above.

**Table 4:** Demographic characteristics of participants (*N* = 547).

Variable	Category	*N* (%)
Gender	Female	427 (78.1%)
	Male	120 (21.9%)
Age	41–54 years	102 (18.6%)
	55–64 years	367 (67.1%)
	65 years and above	78 (14.3%)
Education	Junior high school or below	30 (5.5%)
	Vocational or secondary school	93 (17.0%)
	College diploma	134 (24.5%)
	Bachelor’s degree	194 (35.5%)
	Master’s degree	42 (7.7%)
	Doctorate and above	32 (5.9%)
	Other	22 (4.0%)

**Table 5:** Construct reliability.

Constructs	Variables	SFL	S.E.	*p*	CR	AVE
ER	Er3	0.814	—	—	0.904	0.759
	Er2	0.891	0.046	***		
	Er1	0.906	0.045	***		
AES	Aes4	0.801	—	—	0.891	0.672
	Aes3	0.789	0.049	***		
	Aes2	0.783	0.052	***		
	Aes1	0.901	0.049	***		
MOT	Mot1	0.878	—	—	0.863	0.679
	Mot2	0.843	0.042	***		
	Mot3	0.745	0.045	***		
FLO	Flo1	0.848	—	—	0.937	0.790
	Flo2	0.918	0.042	***		
	Flo3	0.928	0.036	***		
	Flo4	0.858	0.036	***		

Note: ER = Emotional Regulation Function; AES = Aesthetic and Artistic Engagement; MOT = Intrinsic Motivation; FLO = Flow. SFL = standardized factor loading. S.E. is the standard error of the corresponding unstandardized loading from AMOS maximum-likelihood estimation. The first listed indicator of each factor was fixed to 1 for identification, so S.E. is not reported for that item. *** *p* < 0.001.

**Table 6:** Structural path coefficients.

Hypothesis	Path	Std.	Unstd.	S.E.	C.R.	*p*
H1	ER → MOT	0.562	0.552	0.044	12.623	***
H2	AES → MOT	0.256	0.237	0.038	6.250	***
H3	MOT → FLO	0.283	0.389	0.082	4.747	***
H4	ER → FLO	0.228	0.308	0.074	4.169	***
H5	AES → FLO	0.171	0.218	0.057	3.846	***

Note: ER = Emotional Regulation Function; AES = Aesthetic and Artistic Engagement; MOT = Intrinsic Motivation; FLO = Flow. C.R. = critical ratio (unstandardized estimate divided by S.E.), as reported by AMOS; under maximum likelihood this ratio is evaluated as a z statistic. *** *p* < 0.001.

**Table 7:** Bootstrap indirect effects.

Path	Indirect	95% CI		Indirect	95% CI	
	(Unstd.)	Lower	Upper	(Std.)	Lower	Upper
ER → MOT → FLO	0.215	0.121	0.318	0.159	0.090	0.235
AES → MOT → FLO	0.092	0.048	0.148	0.072	0.038	0.114

Note: ER = Emotional Regulation Function; AES = Aesthetic and Artistic Engagement; MOT = Intrinsic Motivation; FLO = Flow.

## Data Availability

The data presented in this study are available on reasonable request from the corresponding author. The data are not publicly available because participants did not provide consent for public archiving of their anonymized responses.

## Supplementary Materials

The following supporting information can be downloaded at: https://www.mdpi.com/article/10.3390/bs16101842/s1, Supplementary Table S1: Scale Items, Construct Sources, and Adaptations; Supplementary Table S2: Age-band SEM feasibility; Supplementary Table S3: Demographic tests on the four constructs; Supplementary Table S4: Item-deletion sensitivity models.

## Abbreviations

The following abbreviations are used in this manuscript:

ER	Emotional Regulation Function
AES	Aesthetic and Artistic Engagement
MOT	Intrinsic Motivation
FLO	Flow
SEM	Structural Equation Modeling
CFA	Confirmatory Factor Analysis
CR	Composite Reliability
AVE	Average Variance Extracted
GFI	Goodness-of-Fit Index
CFI	Comparative Fit Index
IFI	Incremental Fit Index
NFI	Normed Fit Index
TLI	Tucker–Lewis Index
SRMR	Standardized Root Mean Square Residual
RMSEA	Root Mean Square Error of Approximation
AMS	Academic Motivation Scale
MUSE	Music USE Questionnaire

## References

[B1-behavsci-16-01842] Appelgren A., Osika W., Theorell T., Madison G., Bojner Horwitz E. (2019). Tuning in on motivation: Differences between non-musicians, amateurs, and professional musicians. Psychology of Music.

[B2-behavsci-16-01842] Bloska J., Godier-McBard L., Odell-Miller H., Creamer A., Merchant H., Fossey M. (2025). Expanding access to adult community choirs: A scoping review to identify facilitators and barriers to choir membership. Music Education Research.

[B3-behavsci-16-01842] Bonneville-Roussy A., Evans P. (2025). The support of autonomy, motivation, and music practice in university music students: A self-determination theory perspective. Psychology of Music.

[B4-behavsci-16-01842] Browne M. W., Cudeck R. (1992). Alternative ways of assessing model fit. Sociological Methods & Research.

[B5-behavsci-16-01842] Carstensen L. L. (1992). Social and emotional patterns in adulthood: Support for socioemotional selectivity theory. Psychology and Aging.

[B6-behavsci-16-01842] Carstensen L. L. (2006). The influence of a sense of time on human development. Science.

[B7-behavsci-16-01842] Chin T.-C., Rickard N. S. (2012). The Music USE (MUSE) questionnaire: An instrument to measure engagement in music. Music Perception.

[B8-behavsci-16-01842] Csikszentmihalyi M. (1990). Flow: The psychology of optimal experience.

[B9-behavsci-16-01842] Csikszentmihalyi M. (2014). Flow and the foundations of positive psychology: The collected works of Mihaly Csikszentmihalyi.

[B10-behavsci-16-01842] Deci E. L., Ryan R. M. (2000). The “what” and “why” of goal pursuits: Human needs and the self-determination of behavior. Psychological Inquiry.

[B11-behavsci-16-01842] Dillman D. A. (2011). Mail and internet surveys: The tailored design method—2007 update with new internet, visual, and mixed-mode guide.

[B12-behavsci-16-01842] Doscher B. M. (1994). The functional unity of the singing voice.

[B13-behavsci-16-01842] Engeser S., Schiepe-Tiska A., Engeser S. (2012). Historical lines and an overview of current research on flow. Advances in flow research.

[B14-behavsci-16-01842] Evans P., Liu M. Y. (2019). Psychological needs and motivational outcomes in a high school orchestra program. Journal of Research in Music Education.

[B15-behavsci-16-01842] Fornell C., Larcker D. F. (1981). Evaluating structural equation models with unobservable variables and measurement error. Journal of Marketing Research.

[B16-behavsci-16-01842] Gaggioli A., Chirico A., Mazzoni E., Milani L., Riva G. (2017). Networked flow in musical bands. Psychology of Music.

[B17-behavsci-16-01842] Guangming District Cultural Center (2026). 2026 middle-aged and older choir rehearsal.

[B18-behavsci-16-01842] Hair J. F., Black W. C., Babin B. J., Anderson R. E. (2013). Multivariate data analysis *(Pearson New International Edition)*.

[B19-behavsci-16-01842] Hallam S., Creech A. (2016). Can active music making promote health and well-being in older citizens? Findings of the music for life project. London Journal of Primary Care.

[B20-behavsci-16-01842] Hargreaves D. J. (2012). Musical imagination: Perception and production, beauty and creativity. Psychology of Music.

[B21-behavsci-16-01842] Hays T., Minichiello V. (2005). The meaning of music in the lives of older people: A qualitative study. Psychology of Music.

[B22-behavsci-16-01842] Hunan Provincial People’s Government (2024). Huang Donghong interprets the 2023 statistical bulletin on the development of aging affairs in Hunan Province.

[B23-behavsci-16-01842] Jackson S. A., Marsh H. W. (1996). Development and validation of a scale to measure optimal experience: The flow state scale. Journal of Sport and Exercise Psychology.

[B24-behavsci-16-01842] Jang H., Kim E. J., Reeve J. (2016). Why students become more engaged or more disengaged during the semester: A self-determination theory dual-process model. Learning and Instruction.

[B25-behavsci-16-01842] Joseph D., Southcott J. (2018). Music participation for older people: Five choirs in Victoria, Australia. Research Studies in Music Education.

[B26-behavsci-16-01842] Juslin P. N., Västfjäll D. (2008). Emotional responses to music: The need to consider underlying mechanisms. Behavioral and Brain Sciences.

[B27-behavsci-16-01842] Kline R. B. (2023). Principles and practice of structural equation modeling.

[B28-behavsci-16-01842] Laukka P. (2007). Uses of music and psychological well-being among the elderly. Journal of Happiness Studies.

[B29-behavsci-16-01842] Loepthien T., Leipold B. (2022). Flow in music performance and music-listening: Differences in intensity, predictors, and the relationship between flow and subjective well-being. Psychology of Music.

[B30-behavsci-16-01842] Lull J., Lull J. (1987). Listeners’ communicative uses of popular music. Popular music and communication.

[B31-behavsci-16-01842] MacIntyre P. D., Schnare B., Ross J. (2018). Self-determination theory and motivation for music. Psychology of Music.

[B32-behavsci-16-01842] Menninghaus W., Wagner V., Wassiliwizky E., Schindler I., Hanich J., Jacobsen T., Koelsch S. (2019). What are aesthetic emotions?. Psychological Review.

[B33-behavsci-16-01842] Ministry of Culture of the People’s Republic of China (2017). Notice on holding “Forever Glorious”—The 19th China older adults choir festival.

[B34-behavsci-16-01842] Podsakoff P. M., MacKenzie S. B., Lee J.-Y., Podsakoff N. P. (2003). Common method biases in behavioral research: A critical review of the literature and recommended remedies. Journal of Applied Psychology.

[B35-behavsci-16-01842] Preacher K. J., Hayes A. F. (2008). Asymptotic and resampling strategies for assessing and comparing indirect effects in multiple mediator models. Behavior Research Methods.

[B36-behavsci-16-01842] Rentfrow P. J., Gosling S. D. (2003). The do re mi’s of everyday life: The structure and personality correlates of music preferences. Journal of Personality and Social Psychology.

[B37-behavsci-16-01842] Ruud E. (1997). Music and identity. Nordic Journal of Music Therapy.

[B50-behavsci-16-01842] Saarikallio S., Erkkilä J. (2007). The role of music in adolescents’ mood regulation. Psychology of Music.

[B38-behavsci-16-01842] Schäfer T., Sedlmeier P., Städtler C., Huron D. (2013). The psychological functions of music listening. Frontiers in Psychology.

[B39-behavsci-16-01842] Scherer K. R. (2004). Which emotions can be induced by music? What are the underlying mechanisms? And how can we measure them?. Journal of New Music Research.

[B40-behavsci-16-01842] Scherer K. R., Zentner M. R., Juslin P. N., Sloboda J. A. (2001). Emotional effects of music: Production rules. Music and emotion: Theory and research.

[B41-behavsci-16-01842] Siltainsuu H., Peltola H. R. (2024). Music as support for older adults’ wellbeing: A scoping review. Music & Science.

[B42-behavsci-16-01842] Su P., Jiang K., Kong J. (2025). Flow in choral singing: Associations with perceived choral memory performance and well-being among older adults. BMC Psychology.

[B43-behavsci-16-01842] Timmers R., Bannister S., Lennie T. M., Bogunović B., Timmers R., Nikolić S. (2024). Aesthetic emotions in music: Theory, measurements, and cross-cultural comparison. Psychological perspectives on musical experiences and skills: Research in the Western Balkans and Western Europe.

[B44-behavsci-16-01842] Valenzuela R., Codina N., Pestana J. V. (2018). Self-determination theory applied to flow in conservatoire music practice: The roles of perceived autonomy and competence, and autonomous and controlled motivation. Psychology of Music.

[B45-behavsci-16-01842] Vallerand R. J., Pelletier L. G., Blais M. R., Briere N. M., Senecal C., Vallieres E. F. (1992). The academic motivation scale: A measure of intrinsic, extrinsic, and amotivation in education. Educational and Psychological Measurement.

[B46-behavsci-16-01842] Wells A., Hakanen E. A. (1991). The emotional use of popular music by adolescents. Journalism Quarterly.

[B47-behavsci-16-01842] Witherspoon H. (1925). Singing: A treatise for teachers and students.

[B48-behavsci-16-01842] Yao M., Xu Y. (2024). Method bias mechanisms and procedural remedies. Sociological Methods & Research.

[B49-behavsci-16-01842] Zentner M., Grandjean D., Scherer K. R. (2008). Emotions evoked by the sound of music: Characterization, classification, and measurement. Emotion.

