# Supplementary material for: Emotional Regulation, Aesthetic Engagement, and Flow Experience Among Chinese Middle-Aged and Older Choir Members
Source: Behav Sci (Basel). 2026 Oct 8;16(10):1842. doi: 10.3390/bs16101842 (PMC13701255; doi:10.3390/bs16101842)
Supplement: Supplementary file 1 [file behavsci-16-01842-s001.zip › behavsci-4486713-supplementary.pdf]

Supplementary Materials

Supplementary Table S1: Scale Items, Construct Sources, and Adaptations

The questionnaire was administered in Chinese. Supplementary Table S1 presents the Chinese items used in the survey alongside the English wording reported in the manuscript, with the source and adaptation status specified for each item. The English column distinguishes wording retained from a source instrument, wording adapted for the musical context, and researcher-developed wording. Aes3 and Aes4 were newly written. The three motivation items were adapted from the Academic Motivation Scale to musical participation. Flow items follow the simplified four-indicator method of Su et al. (2025), based on the Flow State Scale (Jackson & Marsh, 1996). The Chinese items were translated and adapted jointly by a researcher and a research assistant, both of whom work in music research and read the source instruments in English. The Chinese items are adaptations and are not literal translations of the English source wording. Before distribution, a retired professor of choral music completed the questionnaire and commented on the wording, and the Chinese items were revised accordingly. The same two people then translated the revised Chinese items back into English and compared the result with the source wording. No record was retained of the wording changes or of the back-translation. Cognitive interviewing with members of the target population was not conducted.

Instructions given to participants: the survey was anonymous and for academic research only; there were no right or wrong answers; completion took about 6–8 minutes; unless a question specified otherwise, each item used a 1–5 scale (1 = strongly disagree, 5 = strongly agree); music-function items were to be answered with reference to feelings during choir rehearsal or performance（请结合您在合唱团排练或演唱中的感受作答）.

Music-function items combined adapted MUSE items (Chin & Rickard, 2012) with researcher-developed items. Intrinsic motivation items were adapted from the AMS (Vallerand et al., 1992). Flow items followed the simplified four-indicator method of Su et al. (2025), based on the Flow State Scale (Jackson & Marsh, 1996). Confirmatory factor analysis in the present sample (N = 547) supported the intended structures. AES is labelled aesthetic and artistic engagement.

Item	Administered Chinese	English wording	Original source	Adaptation note
**ER1**	音乐帮助我释放紧张感。	"Music helps me release tension."	MUSE - Cognitive and Emotional Regulation style	Adapted from MUSE ("Music often takes away tension at the end of the day"). Wording simplified, with responses anchored to the choral context.
**ER2**	音乐为情绪提供了一个宣泄的出口。	"Music provides an outlet for emotions."	MUSE - Cognitive and Emotional Regulation style	Adapted from the MUSE Cognitive and Emotional Regulation style. The English wording does not reproduce a specific source item, and responses were anchored to the choral context.
**ER3**	表演合唱对我来说有情感上的回报。	"Performing music is emotionally rewarding for me."	MUSE - Engaged Production style	Adapted from MUSE (Engaged Production) and classified under emotional regulation. English retained from the source instrument. The administered Chinese specifies choral performance.
**Aes1**	音乐能激发我的灵感。	"Music inspires me."	MUSE - inspiration item	Adapted from MUSE ("Music is often a source of inspiration for me"). Wording simplified.
**Aes2**	音乐帮助我思考。	"Music helps me think."	MUSE - reflective engagement item	Adapted from MUSE ("Certain type of music helps me think"). Wording simplified.

**Aes3**	我喜欢特定风格的音乐。	"I like specific styles of music."	Researcher-developed	Researcher-developed. Newly written to cover stylistic preference, which is not in the original MUSE pool.
**Aes4**	我努力精进自己的音乐技巧。	"I strive to master musical techniques."	Researcher-developed	Researcher-developed. Newly written to cover technical-mastery striving, which is not in the original MUSE pool.
**Mot1**	我通过参与音乐活动获取知识。	"I seek knowledge through musical participation."	AMS (Vallerand et al., 1992) - to know	Adapted from the AMS (to know) for the musical context.
**Mot2**	音乐让我感受到满足感。	"I feel fulfilled through music."	AMS (Vallerand et al., 1992) - toward accomplishment	Adapted from the AMS (toward accomplishment) for the musical context.
**Mot3**	歌唱带给我极大的快乐。	"Singing gives me great pleasure."	AMS (Vallerand et al., 1992) - to experience stimulation	Adapted from the AMS (to experience stimulation) for the musical context.
**Flo1**	完全专注于歌唱	"completely focused on singing"	Flow State Scale (Jackson & Marsh, 1996), consolidated per Su et al. (2025)	Independent source: Flow State Scale (Jackson & Marsh, 1996), simplified following Su et al. (2025). Wording made more specific and easier to understand. Precondition dimensions treated as antecedents, not as flow itself.
**Flo2**	忘记了时间的流逝	"losing track of time"	Flow State Scale (Jackson & Marsh, 1996), consolidated per Su et al. (2025)	Independent source: Flow State Scale (Jackson & Marsh, 1996), simplified following Su et al. (2025). Wording made more specific and easier to understand.
**Flo3**	能够掌控整个表演	"being in control of the performance"	Flow State Scale (Jackson & Marsh, 1996), consolidated per Su et al. (2025)	Independent source: Flow State Scale (Jackson & Marsh, 1996), simplified following Su et al. (2025). Wording made more specific and easier to understand.
**Flo4**	觉得歌唱是一件愉悦的事	"finding singing enjoyable"	Flow State Scale (Jackson & Marsh, 1996), consolidated per Su et al. (2025)	Independent source: Flow State Scale (Jackson & Marsh, 1996), simplified following Su et al. (2025). Wording made more specific and easier to understand.

Note. ER = emotional regulation function; AES = aesthetic and artistic engagement; MOT = intrinsic motivation; FLO = flow. MUSE = Music USE Questionnaire (Chin & Rickard, 2012). AMS = Academic Motivation Scale (Vallerand et al., 1992). Main study N = 547, retained from 607 responses after the screening described in the manuscript. Standardised factor loadings, CR, and AVE are reported in Table 5 of the manuscript.

Supplementary Table S2: Age-band SEM feasibility

The 14-item SEM has 34 free parameters. Its structural component is saturated relative to the measurement model. A common heuristic is about 10 observations per free parameter. The 41–54 and 65-and-older groups fall below this heuristic, raising concerns about the stability and precision of age-specific SEM estimates. Measurement invariance was not tested. The primary analysis remains the pooled sample (N = 547). Exploratory age-band path estimates are reported only in the response letter, not as citable findings.

**Age band**	**n**	**Free parameters**	**N/q**
41–54	102	34	3.0
55–64	367	34	10.8
65 and older	78	34	2.3
Pooled (primary)	547	34	16.1

Supplementary Table S3: Demographic tests on the four constructs

The 12-cell statistics below correct the original manuscript sentence that education differences were all $p > 0.05$. Flow differed by education, $F(6, 540) = 2.46$, $p = 0.023$, $\eta^2 = 0.027$. An exploratory check after removing the doctoral group ($n = 32$, $M = 2.80$) gave $F(5, 509) = 1.32$, $p = 0.253$. That check was added during revision and does not replace the full-sample result. For these tests, construct scores were the arithmetic mean of their constituent items. SEM analyses used the individual items as indicators of latent constructs.

Comparison	Statistic	p	Effect size
Gender, ER	t(545) = −1.44	0.151	d = −0.15
Gender, AES	t(545) = 0.21	0.830	d = 0.02
Gender, MOT	t(545) = −1.36	0.175	d = −0.14
Gender, FLO	t(545) = −0.79	0.431	d = −0.08
Age, ER	F(2, 544) = 1.12	0.327	η^2 = 0.004
Age, AES	F(2, 544) = 0.23	0.795	η^2 = 0.001
Age, MOT	F(2, 544) = 0.90	0.408	η^2 = 0.003
Age, FLO	F(2, 544) = 1.50	0.225	η^2 = 0.005
Education, ER	F(6, 540) = 0.71	0.641	η^2 = 0.008
Education, AES	F(6, 540) = 0.50	0.807	η^2 = 0.006
Education, MOT	F(6, 540) = 1.33	0.243	η^2 = 0.015
Education, FLO	F(6, 540) = 2.46	0.023	η^2 = 0.027

Supplementary Table S4: Item-deletion sensitivity models

The primary model retains all 14 items and was reported in the original submission. The three item-deletion models (Er3 dropped, Aes4 dropped, and both items dropped) were estimated after review and are reported as post hoc sensitivity analyses. Interval estimates in the primary column match the revised manuscript. Across all four models, the path difference toward intrinsic motivation excluded 0, and the difference between the two functions' direct paths to flow included 0.

Path	Primary, 14 items	Er3 dropped	Aes4 dropped	Both dropped
ER → MOT	0.562 [0.474, 0.643]	0.543 [0.454, 0.627]	0.565 [0.477, 0.647]	0.546 [0.456, 0.630]
AES → MOT	0.256 [0.169, 0.338]	0.247 [0.159, 0.329]	0.248 [0.165, 0.326]	0.237 [0.153, 0.317]
MOT → FLO	0.283 [0.160, 0.401]	0.272 [0.152, 0.385]	0.293 [0.169, 0.410]	0.281 [0.161, 0.394]
ER → FLO	0.228 [0.129, 0.337]	0.257 [0.156, 0.362]	0.228 [0.127, 0.335]	0.257 [0.158, 0.365]
AES → FLO	0.171 [0.082, 0.259]	0.163 [0.075, 0.250]	0.149 [0.062, 0.238]	0.140 [0.052, 0.228]
Indirect ER	0.159 [0.090, 0.235]	0.148 [0.085, 0.219]	0.165 [0.099, 0.242]	0.153 [0.090, 0.225]
Indirect AES	0.072 [0.038, 0.114]	0.067 [0.035, 0.107]	0.073 [0.040, 0.115]	0.067 [0.035, 0.107]
$\Delta\beta$ to MOT	0.306 [0.163, 0.448]	0.296 [0.145, 0.449]	0.317 [0.175, 0.462]	0.308 [0.162, 0.457]
$\Delta\beta$ to FLO	0.057 [−0.075, 0.191]	0.094 [−0.042, 0.234]	0.078 [−0.053, 0.212]	0.117 [−0.017, 0.260]
R^2 MOT / FLO	0.448 / 0.297	0.425 / 0.307	0.444 / 0.291	0.421 / 0.301
CFI / RMSEA	0.959 / 0.078	0.959 / 0.081	0.960 / 0.080	0.961 / 0.083

Note. H1–H7 remained significant in each sensitivity model. These three deletion models are post-review analyses; they do not replace the 14-item primary model. Values are standardized estimates [bias-corrected 95% confidence intervals] based on 5000 bootstrap resamples (N = 547).
